# Seasonal Patterns of Infectious Diseases

**DOI:** 10.1371/journal.pmed.0020005

**Published:** 2005-01-25

**Authors:** Mercedes Pascual, Andy Dobson

## Abstract

Why is that many infectious diseases, like cholera, malaria, and meningococcal meningitis, show seasonal patterns? And how can we accurately determine these patterns?

Meningococcal meningitis in western Africa shows recurrent seasonal patterns every year. Epidemics typically start at the beginning of February and last until May. We can try to explain the observed patterns on the basis of some seasonally varying environmental factor that favors disease transmission. Air dryness produced by strong dust winds is the most likely candidate.

But while there are qualitative “stories” of this kind in the literature for many seasonal phenomena, convincing quantitative evidence to support them remains largely elusive. Instead, we tend to see weak associations between environmental and transmission variables when measured by simple, linear correlations. The study of meningococcal meningitis in Mali by Sultan and colleagues in this issue of *PLoS Medicine* is a remarkable exception [1]. The study reports a strong association between the yearly onset of epidemics and a large-scale regional index for atmospheric circulation related to the Harmattan winds in Sahelo-Sudanian Africa.

## The Importance of Seasonality

Why is a focus on the seasonality of infectious diseases and its variation from year to year so important? Isn't it more important for us to instead understand the effects of long-term climate change on human health?

At first sight, understanding seasonal patterns seems disconnected from understanding the impact of long-term climate change. However, seasonal patterns are one major pathway for the subtle but potentially drastic effects of climate change on disease dynamics. Long-term climate change affects seasonal patterns through the lengthening of the transmission season and the crossing of environmental and demographic thresholds that underlie seasonal outbreaks [2]. Thus, identifying the specific environmental factors underlying seasonal transmission is a critical step towards predicting and understanding how long-term environmental trends in mean climate and their variability will impact human health.

## The Problem of Scale

One important difficulty in uncovering seasonal drivers of infectious diseases is to identify the appropriate scale of analysis. The relationship between disease and climate described by Sultan and colleagues only becomes apparent at large spatial scales. The authors argue that these large scales are necessary to eliminate “idiosyncratic” variability in the relationship between cases and climate at the local level. In other words, there are only weak correlations between seasonal variations and climate variables at small scales because of the multiple other factors that play a local role and act as noise.

But we should be cautious about the suggestion that appropriate larger scales will always resolve the problem of local variability and present strong linear associations between climate and disease. Public health measures might require predictions not only at national and regional scales, but also at a variety of smaller scales.

Moreover, one important source of variation in how infectious diseases respond to climate is the fraction of susceptible individuals in the population. This fraction varies over time as the result of immunity acquired by previous infection, and by the input of births and migrants into the pool of susceptible people. The constant waxing and waning of this pool of hosts underlies the intrinsic potential of the population dynamics of infectious diseases to oscillate and create epidemic outbreaks. The tendency of these intrinsic cycles to go up and down in synchrony at different locations in space will determine whether susceptibility levels act as noise at small scales or, alternatively, whether their effect must be considered in conjunction with climate at larger scales. Because the number of susceptible individuals is a hidden variable in most epidemiological analyses, recently proposed methods for its reconstruction from data on cases must be combined with studies on climate variation if we are to understand the interaction between susceptibility levels and climate variation [3,4,5].

The problem of scale also arises when we need to identify the appropriate timing (the temporal window) to detect strong associations between disease outbreaks and environmental covariates. This is particularly important when strong couplings between environment and transmission occur only transiently. This seems to be the case for cholera in Bangladesh, where couplings are strong during El Niños, but considerably weaker the rest of the time [6]. Intermittent couplings provide insight into how the system might behave if pushed into specific dynamic regions by a change in climate. Intermittent couplings also suggest the existence of thresholds in the response to climate, an area of research that remains in need of quantitative approaches.

## Seasonal Drivers May Be Elusive

**Figure 1:**
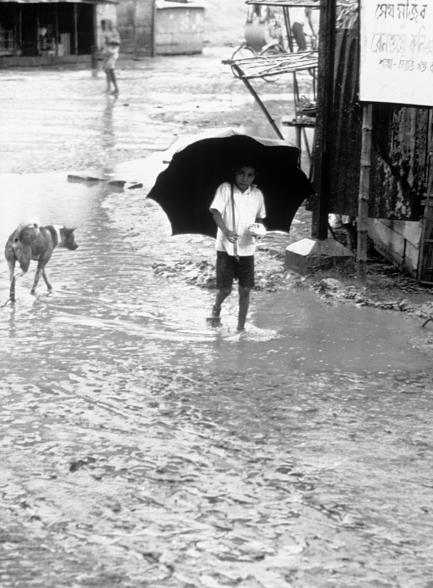
The Role of Rainfall in Driving the Seasonal Nature of Cholera Is Unclear This photograph was taken during a cholera and nutrition survey during flooding in Bangladesh in 1974. In Bangladesh, monsoon rains appear to have a seasonal “dilution” effect on transmission, producing a decrease in cholera cases during that season. We don't know whether extreme rains also produce a lagged increase in cases later on in the cycle. In other parts of the world, cases typically peak during the rainy season. (Photo: Jack Weissman, Centers for Disease Control and Prevention)

Besides scale, specific seasonal drivers are often elusive because of the simpler reason that in nature seasonality is ubiquitous. Multiple and covarying drivers have been proposed for the seasonal nature of cholera, including temperature, rainfall, and plankton blooms [7]. Yet the specific roles of these drivers in the bimodal seasonal cycle of cholera, and particularly in the second peak in endemic regions in south Asia, have not been convincingly shown (Figure 1) [8]. We still don't have predictive explanations of the geographic variation in seasonal patterns. We won't find such explanations by considering the average seasonal pattern; instead, we must consider the anomalies in amplitude and onset of the peaks that occur in different years.

Ecologists have considered seasonality in mathematical models of the population dynamics of infectious diseases. Models of populations with seasonally forced, dynamic interactions (births, deaths, aggregation, or disease transmission) reveal an array of possible responses, from simple yearly cycles, through cycles that repeat with longer periods, to irregular chaotic fluctuations. Some models also predict intermittent switching between different dynamic infectious disease behaviors. But typical models consider only simple seasonal forcing functions (mathematical functions that are periodic in time and therefore describe in a generic way the seasonal variation in the transmission rate or some other seasonal parameter—a sine wave is an example). There are some important exceptions to this—some models do incorporate more complicated seasonal forcing functions that describe the actual processes underlying the seasonal drivers of transmission. Examples are models of childhood diseases that describe the regular stopping and starting of school terms [9,10,11], and recent malaria models that include the seasonal dynamics of mosquito births and pathogen incubation as functions of temperature and rainfall [12].

The explicit way in which models treat seasonal environmental drivers may be critical in addressing the links between within-year seasonal cycles and those of longer period that are observed in many infectious diseases. For meningococcal meningitis, we still need to examine the connection between the seasonal association described by Sultan and colleagues and the previously proposed role of humidity in inter-annual cycles [13].

## The Complexity of Infectious Disease Dynamics

Sultan and colleagues' study is exceptional in that it illustrates a clear relationship between an external environmental variable and the initiation of disease outbreaks. In contrast, many studies seeking environmental drivers are plagued by the many confounding factors, particularly the impact that other components of global change have on the transmission dynamics of infectious diseases. Thus, when we examine datasets for malaria, we must also consider the evolution of drug resistance and a growing human population that is increasingly forced to live in areas that are marginal for agricultural production but optimal for malaria transmission.

Given this complexity, a serious limiting factor to quantitative analyses and predictive models of ecological and disease patterns is the lack of long-term disease records with similar data collected over a network of spatial locations. The handful of extremely valuable records that have allowed progress in understanding long-term patterns in disease dynamics pale in comparison to the spatiotemporal coverage available for climate studies and modeling. The need to resolve these issues of scale and confounding variability only underscores the urgency and importance of maintaining and developing systematic surveillance programs for infectious diseases around the world.
